# The Predictive Validity and Clinical Application of Stopping Elderly Accidents, Deaths & Injuries (STEADI) for Fall Risk Screening

**DOI:** 10.20900/agmr20220008

**Published:** 2022-09-30

**Authors:** Chia-Cheng Lin, Stacey Meardon, Kevin O’Brien

**Affiliations:** 1 Department of Physical Therapy, East Carolina University, Greenville, NC 27834, USA; 2 Department of Biostatistics, East Carolina University, Greenville, NC 27834, USA

**Keywords:** STEADI, fall risk screening, fall prevention

## Abstract

Fall prevention is critical for older adults. Stopping Elderly Accidents, Deaths, and Injuries (STEADI) is a fall prevention initiative, promoted by the Center for Disease Control (CDC). The purpose of this review aims to discuss the predictive validity, improve the predictive validity of STEADI, and apply STEADI in clinical settings.

## INTRODUCTION

The older adult comprises ~16.8% of the United States population and is estimated to be 21.6% in 2040 [1]. With the aging population, health care expenditures will continue to rise. According to the report by the Centers for Medicare & Medicaid Services, it is estimated to increase 7.2% in Medicare and 5.6% in Medicaid spending over 2021–2030 [2], which is 19.6% of the gross domestic product (GDP) [3]. Among the health care expenditures in Medicare, fatal and non-fatal falls among adults aged 65 and older increased from $19 billion in 2007–2009 [4] to $28.9 billion in 2015 [5]. Older adults who fall may suffer pain, functional decline, and hospitalization, in which the medical expenses increases, and decrease the quality of life [6,7]. Thus, identifying individuals at risk for falls is imperative for early intervention to minimize health care costs and optimize the quality of life.

### Factors Associated with the Risk of Falls

Several factors, both unmodifiable and potentially modifiable, may contribute to a greater risk of falling in older adults [8–13]. Unmodifiable factors, include demographics [14] age-related declines [15], history of falls [16,17], and cognitive function [18]. Potentially modifiable factors are associated with health, functional conditions, and environment, including postural hypotension [19], depression [14], vision impairment [20], sensation loss in the feet [21,22], fear of falling [23], neuromuscular and musculoskeletal disorders [24–26], polypharmacy [26,27], and environmental aspects [28,29]. A comprehensive fall prevention approach needs to take both unmodifiable and potentially modifiable factors into account so that the likelihood of falling can be reduced [30–33].

### Stopping Elderly Accidents, Deaths & Injuries (STEADI)

The Center for Disease Control (CDC) has promoted a fall prevention initiative to reduce falls in older adults, called Stopping Elderly Accidents, Deaths, and Injuries (STEADI), developed by the CDC based on the American Geriatrics Society(AGS) and British Geriatrics Society (BGS) guidelines for fall prevention [31,32]. The STEADI considers both unmodifiable and potentially modifiable factors of fall risks and is designed to provide a comprehensive plan of care for individuals for fall risk after assessing by healthcare providers [31]. Stevens and Phelan developed the original STEADI Fall Prevention Toolkit for healthcare providers in 2013 [32]. The original toolkit included instructions and recommendations for fall prevention. The STEADI program was updated in 2019 to include three core elements: Screen, Assess, and Intervene [34]. To screen patients for fall risk, the updated STEADI algorithm (available from https://www.cdc.gov/steadi/pdf/STEADI-Algorithm-508.pdf) contains a 12-question tool (available from https://www.cdc.gov/steadi/pdf/STEADI-Brochure-StayIndependent-508.pdf) with 3-key questions: (1) feeling unsteady, (2) being worried about falling, and (3) recent fall history. If the patients who score more than four points in the 12-question tool or answer “Yes” to any of the three key questions are categorized as Screened At Risk and required further fall risk assessment, otherwise, the patients will be categorized as Screened Not At Risk and no more assessment is needed. The fall risk assessment includes gait, strength, & balance evaluations, medication review, home safety assessment, orthostatic blood pressure testing, and examination of visual acuity, feet/footwear, vitamin D intake, and comorbidities associated with fall risks. A comprehensive list of recommended interventions is provided by the STEADI algorithm and patient-specific recommendations are determined by the assessment findings. For example, medication review and optimization to reduce fall risk are recommended for patients with polypharmacy, and a physical therapy referral for gait, strength, and balance training is recommended for patients with deficits in these areas. A follow-up visit is also recommended within 30–90 days to improve the care plan and address the barriers to fall prevention. STEADI-Rx, which focuses on medication review, is also developed to improve collaboration between pharmacists and healthcare providers and is included in the 2019 update. Overall, STEADI promotes interdisciplinary collaboration for fall prevention in the clinical setting.

The purpose of this review was to discuss the predictive validity, improve the predictive validity of STEADI, and apply STEADI in different clinical settings.

## SEARCH STRATEGY

The keyword “STEADI” and “Fall” was used to search in PubMed.gov on September 21, 2022. A total of Fifty-six studies were identified with the keywords. The inclusion criteria were (1) studies investigating the predictive validity of STEADI, (2) studies aimed to improve the predictive validity for STEADI, and (3) studies utilizing STEADI for fall prevention with a research design and supplying data analysis. Twenty-two manuscripts were found implementing STEADI with a study design and supplied a consequential data analysis. Among the twenty-one papers, four examined the predictive validity of STEADI, six studies addressed improving the predictive validity of STEADI, and twelve investigated implementing STEADI in clinical practice, healthcare education, community-based pharmacy, or community-based fall screening program.

## DISCUSSION

### The Predictive Validity of STEADI

The predictive validity of a screening tool indicates how well the screening tool can identify people with a certain condition, i.e., falls. A fall screening tool with better predictive validity will be able to pick up older who had a fall history or will fall in the future. Three studies investigated the predictive validity of STEADI [35–37] and one study did not examine STEADI directly but investigated the 3-key question and 12-question tool for fall screening [38]. Table 1 summarized the prospective and retrospective predictive value for STEADI, 3-key question, and 12-question tool. Nithman and Vincenzo reported STEADI algorithm was able to differentiate between older adults with a history of falls in the past 12 months with a sensitivity of 68.6% and specificity of 47.6% and predict prospective falls in 6 months with a sensitivity of 68.4% and specificity of 44.9% for a combined sample (*N* = 77) from retirement center and community-dwelling older adults [36]. However, the STEADI demonstrated better predictive validity in community-dwelling older adults (*n* = 39; Sensitivity 73–80%) compared with the retirement facility-dwelling older adults (*n* = 38; Sensitivity 56–62%) [36]. Another study by Loonlawong et al., translated the 12-question tool and 3-key questions into the Thai language and applied the translated STEADI algorithm to the six local hospitals [37]. After a 12-month follow-up, they found 3-key questions with a sensitivity of 93.9% and specificity of 75% (AUG: 0.85) and the translated 12-question tool with a sensitivity of 77.7% and specificity of 88.0% (AUG: 0.83) had good prospective predictive validities. No combined predictive validity (3 key questions + 12-question tool) values were provided in this study [37]. However, Loonlawong et al. stated their overall validity of the STEADI algorithm was higher than those reported previously [37]. It is important to note that the all discussed above studies used the older version of the STEADI algorithm, which classified the fall risk into three levels (lower, moderate, and higher risks). Studies are needed to validate the effectiveness of the updated version of the STEADI algorithm. Burns et al. did not use the STEADI concept but examined the predictive values for falls on the 3-key questions and 12-question tool to compare with other fall screening tools [38]. Their data showed that the 3-key questions had a sensitivity of 68.7% and specificity of 57.9%, and the 12-question tool had a sensitivity of 55.7% and specificity of 75.9% with 11-month follow-up data. Lohman et al. also reported that the predictive validity for the STEADI algorithm was fair with a sensitivity of 65% and specificity of 65% (AUC: 0.64) utilizing retrospective data [35].

### Improving Predictive Validity for STEADI

Six studies were identified that aimed to improve predictive validity for the STEADI algorithm by modifying score calculation [39], changing fall risk levels [40], adding additional supplemental questions [41], frailty status [42], feet/footwear screening tools [43], and performing different physical assessment [44]. Helsel et al. used a point method to find coefficient-based integers to predict 4-year fall risk. Falls in the past year, multiple falls, and fear of falling were identified as significant predictors [39]. Mielenz et al., proposed using a two-level fall risk (at-risk and not at-risk) screening algorithm instead of three-level algorithms [40]. They reported that as two-level STEADI algorithm (AUC: 0.65) had similar predictability for falls compared with three-level STEADI (AUC: 0.66) and would be easier to use in the clinical setting [40]. Sri-on et al. suggested that a STEADI score ≥ 4 was insufficient to predict adverse events after falls, such as death, ED revisits, subsequent hospitalization, or recurrent falls [41]. They recommended adding four supplemental questions: Do you “Use or have been advised to use a cane or walker”, “Take medication that sometimes makes them feel light-headed or more tired than usual ”, Take mediation to help sleep or improve mood”, and “Have to rush to a toilet” to predict the potential adverse event [41]. Wingood et al., proposed an additional feet/footwear screening tool to augment the STEADI [43]. However, no data was available since the feet/footwear screening tool is still under development. Welch et al., suggested that combining the Short Physical Performance Battery (SPPB) with STEADI might help to identify people with negative STEADI fall screen but had a poor physical performance placing them at risk of fall [44]. However, Crow found that adding frailty status did not improve STEADI algorithm to predict falls [42]. Among all of aforementioned studies that suggested improvement for the STEADI, no predictive validity values were provided for the STEADI with the additional screening questions or assessments. The updated STEADI has added the cutoff cores of 4 for the questionnaire and classify the fall risk into two levels (not at risk and at risk).

### Applying STEADI in Different Settings

Studies examining the use of STEADI in primary care settings may provide insightful recommendations during the integration process. Table 2 summarized the recommendations/suggestions when implementing STEADI in different settings. Stevens et al. concluded STEADI can be integrated into a primary care setting with proper staff training, electronic health records (EHR) incorporation, and adapted into clinical workflow [45], while Casey et al. have suggested the key to successful employment of STEADI was using EHR to guide clinic flow and support from clinical champions [46]. Another study by Eckstrom et al. incorporated STEADI into EHR in the Internal Medicine and Geriatrics Clinic and found using 3-key questions might help with the clinic flow but could increase patients categorized with high risk [47]. A study by Johnston et al. revealed that STEADI utilization can reduce fall-related hospitalization and lower medical expenditures [48]. However, when applying STEADI to the primary care setting, the STEADI training material may be not sufficient for the clinical staff, and support from the clinic champions is critically important. Urban et al. were interested in promoting STEADI in the primary care setting and found that written materials and an online model did not increase the overall knowledge and use of the STEADI toolkit [49]. They recommended having a facilitator for STEADI and incorporating with HER [49].

Adding STEADI to the emergency department (ED) may help with identifying older adults with fall risk. Greenberg et al. applied STEADI to ED practice [50]. Their subjects were divided into the control and intervention groups. The care plans to reduce fall risks were provided to the intervention group. However, after a 12-month follow-up, no statistical difference in fall occurrence was found between groups during any point in this study [50]. The obvious disadvantage of applying STEADI into ER practice is the inability to follow up with the patients for their compliance with the interventions [50].

A community-based pharmacy may be a good place to implement STEADI, especially for providing a comprehensive medication review. Hughes et al. applied STEADI in a community-based pharmacy and provided a comprehensive medication review (CMR) on individuals with fall risk [51]. They reported that 52.8% of the providers responded to the faxed SOAP note for CMR intervention, which might suggest the communication gap between the local pharmacy and the providers’ office [51]. Another study utilized the updated STEADI-Rx in the community pharmacy settings [52]. A total of 65 pharmacies joined this study with a total of 10,565 patients [52]. Blalock et al. did not find the difference between control and intervention (STEADI screen with and without Med review) groups in the Drug Burden Index and the risk of falling over time [52]. However, they demonstrated a model to implement STEADI-Rx in a local pharmacy setting.

STEADI has been used in community-based fall risk screening. Karlsson et al. found STEADI toolkit could lead to short- and long-term behavior changes to reduce fall risk when using STEADI as a fall screening tool for community-dwelling older adults [53]. Knight applied the STEADI model in a rural native American community and the participants improved their physical function after the intervention [54]. However, only 17 out of 192 participants completed the intervention [54].

Although CDC has promoted STEADI into clinical practice, a national survey of physical therapists and physical therapist assistants for their knowledge and use of STEADI for fall screening in older adults found that 51% of the sample (*n* = 425) were familiar (*n* = 84) to very familiar (*n* = 132) with STEADI, 21.7% (*n* = 92) were not familiar at all with STEADI in clinical practice, and only 26.1% (*n* = 111) utilized STEADI in clinical practice [56]. To promote the awareness of using STEADI in clinical practice and the nature of interdisciplinary collaboration among healthcare providers, STEADI has been used for interprofessional education for fall prevention. Tayler et al. implemented STEADI for interprofessional education (IPE) and service-learning activities [55]. They found students improved students’ knowledge of fall prevention, STEADI, and their roles in the interdisciplinary team [55]. Encouraging STEADI content in the educational agendas for future healthcare providers may be a good way to promote STEADI.

### Future Research Directions

Studies have examined the predictive validity of STEADI and showed fair sensitivity and poor specificity for prospective fall prediction. However, no large-scale study has been done, especially implementation in clinical practice to investigate the long-term effectiveness of STEADI. Moreover, studies aimed to improve the predictive validity of STEADI may require special training for using the supplement tools. An easy assessment tool may be needed in the clinic for healthcare providers along with STEADI so that the predictive validity of STEADI may be improved. Considering the importance of somatosensation for postural control and sensation screening is lacking in STEADI. Utilizing sensation screen tools, such as monofilament or biothesiometer may help improve the predictive validity of STEADI. Future studies may consider a large-scale study in the clinical setting to validate the effectiveness of STEADI for fall prevention and add an easy-to administrate clinical assessment tool to improve the predictive validity of STEADI. Moreover, the updated STEADI has not been investigated yet.

## CONCLUSIONS

Current evidence suggests that the STEADI displays fair predictive validity for fall prediction. However, utilizing STEADI in different settings may promote fall prevention in community-dwelling older adults. Future large-scale studies may need to examine the effectiveness of STEADI and continue to improve the predictive validity for fall prediction.

## Figures and Tables

**Table 1.** Prospective and retrospective predictive values of STEAD, 3-key question (3KQ), and 12-question tool (12Q).

Author	Year	Tool	Sensitivity	Specificity	Note
**Prospective Falls**					
Nithman and Vincenzo [36]	2019	STEADI	68.4%	44.9%	6-month prospective falls
		3KQ	78.9%	34.7%	
		12Q	52.6%	61.2%	
Loonlawong et al. [37]	2022	STEADI			12-month Prospective falls
		3KQ	93.9%	75%	
		12Q	77.7%	88.0%	
Burns et al. [38]	2022	STEADI			11-month Prospective falls
		3KQ	68.7%	57.9%	
		12Q	55.7%	75.9%	
**Retrospective Falls**					
Lohman et al. [35]	2017	STEADI	65.0%	65.0%	Previous Fall History
		3KQ			
		12Q			
Nithman and Vincenzo [36]	2019	STEADI	68.6%	47.6%	12-month Fall History
		3KQ	100%	50%	
		12Q	71.4%	73.4%	

**Table 2.** Appling STEADI in various settings and recommendations for implementing STEADI.

Author	Setting	Recommendations/Suggestions
Stevens et al. [45]	Primary care setting	Proper staff training, HER incorporation, and support from clinic champions
Casey et al. [46]		using EHR to guide clinic flow and support from clinical champions
Johnston et al. [48]		Proper training material, and support from clinic champions
Urban et al. [49]		Facilitator for STEADI and incorporating with EHR
Eckstrom et al. [47]	Internal Medicine and Geriatrics Clinic	Ask 3-key questions to screen patients’ fall risk
Greenberg et al. [50]	Emergency department	Inability to follow up may need follow-up by PCP
Hughes et al. [51]	Community-based pharmacy	Need to improve the communication between the local pharmacy and the providers’ office
Blalock et al. [52]		Apply STEADI to a local pharmacy is possible
Karlsson et al. [53]	Community fall screening	STEADI helped with fall screening and led to behavior changes in community-dwelling older adults
Knight [54]		Improved participants’ physical function, but the drop-up rate was high
Tayler et al. [55]	IPE education	Encouraging STEADI content in the educational agendas for future healthcare providers

HER: Electronic health records; IPE: Interprofessional education; PCP: primary care physician.

## Data Availability

Data sharing not applicable to this article as no datasets were generated or analyzed during the current study.
